# Ca^2+^ cycling properties are conserved despite bradycardic effects of heart failure in sinoatrial node cells

**DOI:** 10.3389/fphys.2015.00018

**Published:** 2015-02-02

**Authors:** Arie O. Verkerk, Marcel M. G. J. van Borren, Antoni C. G. van Ginneken, Ronald Wilders

**Affiliations:** ^1^Department of Anatomy, Embryology and Physiology, Academic Medical Center, University of AmsterdamAmsterdam, Netherlands; ^2^Laboratory of Clinical Chemistry and Haematology, Rijnstate HospitalArnhem, Netherlands

**Keywords:** heart failure, pacemaker activity, intracellular Ca^2+^, Ca^2+^ clock, membrane clock, sinoatrial node, action potentials, sodium-calcium exchanger

## Abstract

**Background:** In animal models of heart failure (HF), heart rate decreases due to an increase in intrinsic cycle length (CL) of the sinoatrial node (SAN). Pacemaker activity of SAN cells is complex and modulated by the membrane clock, i.e., the ensemble of voltage gated ion channels and electrogenic pumps and exchangers, and the Ca^2+^ clock, i.e., the ensemble of intracellular Ca^2+^ ([Ca^2+^]_i_) dependent processes. HF in SAN cells results in remodeling of the membrane clock, but few studies have examined its effects on [Ca^2+^]_i_ homeostasis.

**Methods:** SAN cells were isolated from control rabbits and rabbits with volume and pressure overload-induced HF. [Ca^2+^]_i_ concentrations, and action potentials (APs) and Na^+^–Ca^2+^ exchange current (I_NCX_) were measured using indo-1 and patch-clamp methodology, respectively.

**Results:** The frequency of spontaneous [Ca^2+^]_i_ transients was significantly lower in HF SAN cells (3.0 ± 0.1 (*n* = 40) vs. 3.4 ± 0.1 Hz (*n* = 45); mean ± SEM), indicating that intrinsic CL was prolonged. HF slowed the [Ca^2+^]_i_ transient decay, which could be explained by the slower frequency and reduced sarcoplasmic reticulum (SR) dependent rate of Ca^2+^ uptake. Other [Ca^2+^]_i_ transient parameters, SR Ca^2+^ content, I_NCX_ density, and I_NCX_-[Ca^2+^]_i_ relationship were all unaffected by HF. Combined AP and [Ca^2+^]_i_ recordings demonstrated that the slower [Ca^2+^]_i_ transient decay in HF SAN cells may result in increased I_NCX_ during the diastolic depolarization, but that this effect is likely counteracted by the HF-induced increase in intracellular Na^+^. β-adrenergic and muscarinic stimulation were not changed in HF SAN cells, except that late diastolic [Ca^2+^]_i_ rise, a prominent feature of the Ca^2+^ clock, is lower during β-adrenergic stimulation.

**Conclusions:** HF SAN cells have a slower [Ca^2+^]_i_ transient decay with limited effects on pacemaker activity. Reduced late diastolic [Ca^2+^]_i_ rise during β-adrenergic stimulation may contribute to an impaired increase in intrinsic frequency in HF SAN cells.

## Introduction

Pacemaker activity of the sinoatrial node (SAN) is controlled by a complex system of clocks composed of time- and voltage-dependent sarcolemmal currents, designated the voltage or membrane clock (Mangoni and Nargeot, 2008), and tightly coupled sarcoplasmic reticulum (SR) Ca^2+^ cycling molecules together with the Na^+^–Ca^2+^ exchange current (I_NCX_), named the Ca^2+^ clock (Lakatta et al., 2010). The primary mechanism of SAN pacemaking is heavily debated (DiFrancesco and Robinson, 2002; Kodama et al., 2002; Vinogradova et al., 2002b; Honjo et al., 2003; Lakatta et al., 2003; Lipsius and Bers, 2003; Bers, 2006a; Lakatta and DiFrancesco, 2009; DiFrancesco, 2010; Maltsev and Lakatta, 2010a, 2012; Verkerk and Wilders, 2010; Himeno et al., 2011a,b; Maltsev et al., 2011; DiFrancesco and Noble, 2012a,b; Lakatta and Maltsev, 2012), but it likely involves a tight collaboration of both clock systems, because intracellular Ca^2+^ ([Ca^2+^]_i_) affects various membrane currents directly, e.g., I_NCX_ (Blaustein and Lederer, 1999), hyperpolarization-activated current (I_f_) (Rigg et al., 2003), slow component of delayed rectifier K^+^ current (I_Ks_) (Tohse, 1990), long lasting Ca^2+^ current (I_Ca,L_) (Sipido et al., 1995), transient Ca^2+^ current (I_Ca,T_) (Lacinová et al., 2006), Cl^−^ current (Arai et al., 1996; Verkerk et al., 2002), and/or indirectly via calmodulin-dependent protein kinase II (CaMKII) (Wu and Anderson, 2014), and changes in membrane current affect pacemaker rate with resultant changes in [Ca^2+^]_i_ (Lakatta et al., 2010; van Borren et al., 2010).

Heart failure (HF) reduces pacemaker activity of SAN cells through an increase in intrinsic cycle length (Opthof et al., 2000; Witte et al., 2000; Verkerk et al., 2003; Du et al., 2007). Previously, it has been shown that this was al least due to remodeling of components of the membrane clock. HF impairs the membrane clock by downregulation of I_f_ in the SAN of rabbit (Verkerk et al., 2003) and downregulation of the corresponding hyperpolarization-activated (HCN) channel subunits, HCN4 and HCN2, in the SAN of dogs (Zicha et al., 2005). In addition, TTX-sensitive neuronal Na^+^ channels, Na_v_1.1 and Na_v_1.6, are downregulated in SAN tissue of HF rats (Du et al., 2007). Finally, HF upregulates I_Ks_ in HF SAN cells of rabbit (Verkerk et al., 2003), but since I_Ks_ plays a limited role in pacemaker activity without adrenergic stimulation (Lei et al., 2000), this change in membrane clock is hardly involved in the increase in intrinsic cycle length during HF (Verkerk et al., 2003).

To date, the effect of HF on [Ca^2+^]_i_ homeostasis in atrial and ventricular myocytes has been studied in detail (for reviews, see Bers, 2006b; Bers et al., 2006; Kho et al., 2012; Eisner et al., 2013; Luo and Anderson, 2013; Neef and Maier, 2013), but the effect of HF on [Ca^2+^]_i_ in SAN cells is hardly known. Shinohara et al. (2010) found that HF, induced by rapid ventricular pacing, results in Ca^2+^ clock malfunction in SAN of dogs, characterized by a reduction of the slope of late diastolic Ca^2+^ elevation (LDCAE) as well as unresponsiveness to isoproterenol and caffeine in intact SAN. Especially the reduced slope of the LDCAE, with associated decrease in localized Ca^2+^ releases (LCRs) or Ca^2+^ sparks (Bogdanov et al., 2006; Maltsev et al., 2006; Joung et al., 2009, 2010; van Borren et al., 2010), may have implications for the Ca^2+^ clock (Stern et al., 2014).

In the present study, we evaluated the [Ca^2+^]_i_ homeostasis in isolated control (CTRL) and HF SAN cells using a well-established rabbit model of volume and pressure overload-induced HF. We found a slower spontaneous [Ca^2+^]_i_ transient frequency in HF SAN cells, indicating that the intrinsic cycle length was prolonged. HF slowed the [Ca^2+^]_i_ transient decay without changes in diastolic and systolic [Ca^2+^]_i_ concentrations, LDCAE, and SR Ca^2+^ content. The reduced [Ca^2+^]_i_ transient decay velocity was partially, but not completely, explained by the slower intrinsic frequency of HF SAN cells. Neither the I_NCX_ density nor the I_NCX_-[Ca^2+^]_i_ relationship were affected by HF. Combined action potential and [Ca^2+^]_i_ measurements demonstrated that the decreased [Ca^2+^]_i_ transient decay velocity has limited effect on I_NCX_ during diastolic depolarization.

## Materials and methods

### Cell preparation

The investigation was approved by the local ethics committee and conformed to the guiding principles of the *Declaration of Helsinki*. HF was induced in 5-month-old male New-Zealand White rabbits by combined volume- and pressure-overload in 2 sequential surgical procedures as described previously (Vermeulen et al., 1994; Verkerk et al., 2011). In short, volume overload was produced by rupture of the aortic valve until pulse pressure was increased by about 60–90%. Three weeks later, pressure overload was created by suprarenal abdominal aorta constriction of approximately 50%. Four months after the last operation, the rabbits were anaesthetized [ketamine (50 mg im; Eurovet, Bladel, The Netherlands) and xylazine (10 mg im; Eurovet, Bladel, The Netherlands)], heparinized (5000 IU, LEO Pharma, Breda, The Netherlands), and killed by intravenous injection of pentobarbital (240 mg; Ceva Sante Animale B.V., Maassluis, The Netherlands). Relative heart weight (i.e., heart weight to body weight ratio), relative lung weight (i.e., lung weight to body weight ratio), and presence of ascites were analyzed as described previously (Vermeulen et al., 1994). Sham-operated rabbits undergoing thoracotomy and age-matched non-operated rabbits do not differ in various heart failure parameters (Vermeulen et al., 1994) and important cellular parameters for hypertrophy and ionic remodeling (Verkerk et al., 2011). In the present study, therefore, non-operated age-matched healthy animals served as control (CTRL). A total of 12 HF and 13 CTRL rabbits were used.

Single SAN cells were enzymatically isolated from the entire SAN region as described previously (Verkerk et al., 2009). Small aliquots of cell suspension were put in a recording chamber on the stage of an inverted microscope. Cells were allowed to adhere for 5 min after which superfusion with Tyrode's solution was started. Tyrode's solution (36 ± 0.2°C) contained (in mM): NaCl 140, KCl 5.4, CaCl_2_ 1.8, MgCl_2_ 1.0, glucose 5.5, HEPES 5.0, pH 7.4 (NaOH). Spindle and elongated spindle-like cells displaying regular contractions were selected for measurements.

### Calcium measurements

[Ca^2+^]_i_ was measured in spontaneously active indo-1 (Molecular Probes, Eugene, OR, USA) loaded myocytes as described previously in detail (van Borren et al., 2010). Such signals are a measure of global [Ca^2+^]_i_ transients, which are triggered by Ca^2+^ influx through sarcolemmal Ca^2+^ channels activated during spontaneous action potentials. As indicated in the Introduction section, these spontaneous action potentials in SAN cells likely involve a tight collaboration of both membrane and Ca^2+^ clock systems. Of note, in this study we used the term “spontaneous [Ca^2+^]_i_ transient” analog to “spontaneous action potentials,” without assumptions regarding the primary cause of the spontaneous activity. As before, we discerned and analyzed five distinct phases in [Ca^2+^]_i_ transients of SAN cells (van Borren et al., 2010): (1) minimum diastolic [Ca^2+^]_i_ concentration, (2) maximum systolic [Ca^2+^]_i_ concentration, (3) maximum rate of the fast systolic [Ca^2+^]_i_ rise, (4) time constant of monoexponential [Ca^2+^]_i_ transient decay, and (5) mean rate of the slow diastolic [Ca^2+^]_i_ rise during the final third of diastolic depolarization until the fast systolic [Ca^2+^]_i_ transient. Ten consecutive spontaneous [Ca^2+^]_i_ transients were used to determine the average parameters. To date, SR Ca^2+^ content and the rate of Ca^2+^ uptake into the SR by sarco/endoplasmic reticulum Ca^2+^-ATPase (SERCA) in cardiac working myocytes are frequently measured using caffeine-induced [Ca^2+^]_i_ transients combined with simultaneous I_NCX_ recordings with the patch clamp methodology (Varro et al., 1993), which also allows specific pre-pacing protocols. In SAN cells, however, we were unable to measure reliably the I_NCX_ during caffeine-induced Ca^2+^ transients, due to cell membrane rupture resulting in large “leak” currents in response to the strong and fast caffeine-induced contractions. Therefore, we assessed SR Ca^2+^ content as the fractional release of SR Ca^2+^ (Bers, 1987; Bassani et al., 1992) by comparing the amplitude of the spontaneous [Ca^2+^]_i_ transients (average of the preceding six successive spontaneous [Ca^2+^]_i_ transients) with that of 20 mM caffeine-evoked [Ca^2+^]_i_ transient in the presence of 5 mM NiCl_2_, to block the I_NCX_. The rate of decay was obtained by fitting single exponential functions to the decay phase of the caffeine-evoked [Ca^2+^]_i_ transients.

### Electrophysiology

Action potentials and I_NCX_ were recorded by the amphotericin-perforated patch-clamp technique using an Axopatch 200B amplifier (Molecular Devices, USA). Patch pipettes (borosilicate glass, 2–5 MΩ) were filled with solution containing (in mM): K-gluc 120, KCl 20, NaCl 5, amphotericin-B 0.22, NMDG-Cl (*N*-methyl-D-glucammonium chloride) 10, HEPES 10, pH 7.2 (KOH 5.5). Series resistance was compensated by ≥80%, and potentials were corrected for the calculated liquid junction potential. Action potentials were low-pass filtered (cut-off frequency: 1 kHz) and digitized at 1 kHz; I_NCX_ was filtered and digitized at 1 and 0.2 kHz, respectively. Voltage control and data acquisition were accomplished using pCLAMP 8 software (Molecular Devices, USA), while custom software was used for data analysis. Cell membrane capacitance was estimated by dividing the time constant of the decay of the capacitive transient in response to 5 mV hyperpolarizing voltage clamp steps from 0 mV by the series resistance, and amounted to 50.0 ± 6.9 pF in CTRL (*n* = 5) and 56.1 ± 8.1 pF in HF (*n* = 5) SAN cells (mean ± SEM, *P* > 0.05), in line with the values of 50 ± 4 (*n* = 23) and 52 ± 3 pF (*n* = 24) that we observed in a previous study (Verkerk et al., 2003). I_NCX_ density and the I_NCX_-[Ca^2+^]_i_ relationship were measured as described previously (Weber et al., 2003). In short, a slow depolarizing voltage ramp from −85 to 120 mV results in an increase in [Ca^2+^]_i_ due to Ca^2+^ entry through NCX operating in reverse mode (net outward I_NCX_). Upon fast repolarization, Ca^2+^ removal occurs resulting in a [Ca^2+^]_i_ decline due to Ca^2+^ efflux through NCX operating in forward mode (net inward I_NCX_) (Weber et al., 2003). The relationship between [Ca^2+^]_i_ and I_NCX_ is membrane potential dependent, with a steeper relationship at more negative membrane potentials (Hove-Madsen and Tort, 2001). We used a repolarizing potential of −60 mV, which is close to the maximal diastolic potential of both CTRL and HF SAN cells (Verkerk et al., 2003). Thapsigargin (2.5 μM) was added to the Tyrode's solution to block SERCA. In addition, chromanol (5 μM) and E4031 (5 μM) were present to block the slow and rapid components of the delayed rectifier K^+^ current, while CsCl (5 mM) was present to block I_f_. All drugs were obtained from Sigma (Zwijndrecht, The Netherlands) except for E4031, which was a gift from Eisai (Tokyo, Japan), and noradrenaline (Centrafarm, Etten-Leur, The Netherlands). I_NCX_ densities were calculated by dividing current amplitudes by the cell membrane capacitance.

### Numerical reconstruction of sodium-calcium exchange current

For the numerical reconstruction of I_NCX_ on the basis of the simultaneously recorded experimental data on the membrane potential (V_m_) and [Ca^2+^]_i_ (combined voltage and calcium clamp, van Borren et al., 2007), we adopted the I_NCX_ formulation of the Lindblad et al. model for a rabbit atrial myocyte (Lindblad et al., 1996), with I_NCX_ scaled down to 78% of its control value, based on mRNA data from Allah et al. (2011). We thus followed the approach of our previous study in which we reconstructed rabbit SAN I_NCX_ in order to compare it with human SAN I_NCX_ (Verkerk et al., 2013).

The Lindblad et al. (1996) equations not only require values for V_m_ and [Ca^2+^]_i_, but also for [Ca^2+^]_e_, [Na^+^]_e_ and [Na^+^]_i_, which denote the extracellular Ca^2+^ concentration, the extracellular Na^+^ concentration, and the intracellular Na^+^ concentration, respectively. [Ca^2+^]_e_ and [Na^+^]_e_ were set to 1.8 and 140 mM, respectively, in accordance with the aforementioned Tyrode's solution, whereas [Na^+^]_i_ was set to 5 mM, based on the pipette solution. In some reconstructions, [Na^+^]_i_ was raised to 7.5 mM, as indicated.

### Statistics

Data are mean ± SEM. Groups of HF SAN cells were compared with groups of CTRL SAN cells using Fisher's exact test, Two-Way Repeated Measures ANOVA followed by pairwise comparison using the Student-Newman-Keuls test, and unpaired *t*-test. Paired *t*-tests were used to compare drug effects within a group. An *F*-test was used to compare frequency dependency between groups. *P* < 0.05 is considered statistically significant.

## Results

### HF model characteristics

Table 1 summarizes the general characteristics of the HF model. Consistent with previous reports of the volume- and pressure-overload rabbit model of HF (Vermeulen et al., 1994; Baartscheer et al., 2003a,b; Verkerk et al., 2003, 2011; Wiegerinck et al., 2006) body weight was similar, but heart weight was significantly higher in HF compared to CTRL rabbits. Consequently relative heart weight was significantly higher in HF rabbits. In addition, HF rabbits had an increased lung weight and relative lung weight. Moreover, six out of the 12 HF rabbits used in the present study exhibited ascites as assessed during autopsy, while none of the 13 CTRL rabbits did. Finally, we measured the weight of the right atrium to determine whether HF affects also the right atrium including the SAN. We found that both the absolute weight of the right atrium and the relative right atrial weight were significantly higher in HF rabbits.

**Table 1:** **Characteristics of the control (CTRL) and heart failure (HF) model**.

	**CTRL (*N* = 13)**	**HF (*N* = 12)**
Body weight (kg)	3.90 ± 0.09	3.92 ± 0.13
Heart weight (g)	9.0 ± 0.4	19.2 ± 1.3^*^
Relative heart weight (g kg^−1^)	2.31 ± 0.08	4.90 ± 0.26^*^
Lung weight (g)	12.2 ± 0.4	18.8 ± 1.3^*^
Relative lung weight (g kg^−1^)	3.13 ± 0.08	4.84 ± 0.35^*^
Presence of ascites (%)	0	50^*^
Right atrium weight (g)	0.37 ± 0.04	0.86 ± 0.16^*^
Relative right atrium weight (g kg^−1^)	0.09 ± 0.01	0.22 ± 0.04^*^

* *P < 0.05 vs. CTRL. N, number of rabbits*.

### HF reduces the frequency and Ca^2+^ transient decay of SAN cells

**Figure 1:**
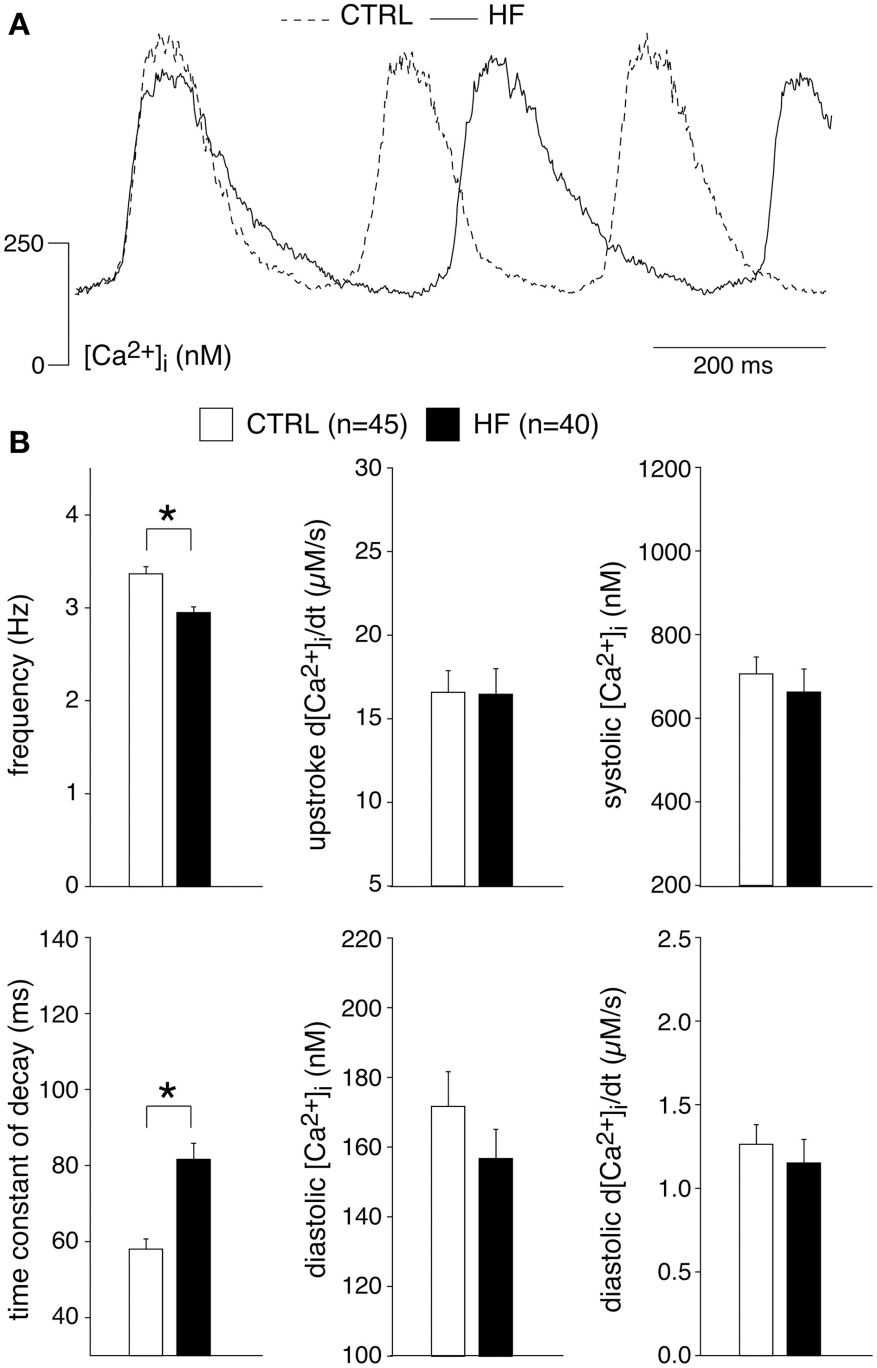
**Heart failure (HF) reduces the frequency and intracellular Ca^2+^ ([Ca^2+^]_i_) transient decay of sinoatrial (SAN) cells**. **(A)** Representative [Ca^2+^]_i_ transients in a SAN cell isolated from a control (CTRL) and HF rabbit. **(B)** Average [Ca^2+^]_i_ transient characteristics. ^*^P < 0.05.

Figure 1A shows typical recordings of [Ca^2+^]_i_ transients from SAN cells of a CTRL and a HF rabbit; Figure 1B summarizes the average [Ca^2+^]_i_ characteristics. In SAN cells, spontaneous action potentials are accompanied by [Ca^2+^]_i_ transients in a 1:1 fashion in CTRL (Li et al., 1997; Rigg et al., 2000; Lakatta et al., 2003; Joung et al., 2009, 2010; van Borren et al., 2010) as well as in HF SAN cells (see **Figure 7**, below). Thus, the rate of spontaneous SAN [Ca^2+^]_i_ transients is a measure of the intrinsic beating rate. We found a significantly lower frequency of spontaneous [Ca^2+^]_i_ transients in HF SAN cells [Figure 1B, top left; 2.95 ± 0.06 (HF) vs. 3.37 ± 0.08 (CTRL) Hz], indicating that intrinsic cycle length was prolonged in HF SAN cells. The upstroke velocity of the [Ca^2+^]_i_ transient and the systolic [Ca^2+^]_i_ transient concentration were not significantly different in HF SAN cells compared with CTRL SAN cells (Figure 1B, top right panels). The decay of the [Ca^2+^]_i_ transient was significantly slowed in HF SAN cells, as indicated by the significant increase in the time constant of decay (Figure 1B, bottom left). On average, [Ca^2+^]_i_ transient time constant of decay [81.6 ± 4.2 (HF) vs. 57.8 ± 2.6 (CTRL) ms] is ≈41% larger than in CTRL SAN cells. Neither the diastolic [Ca^2+^]_i_ concentration nor the late diastolic [Ca^2+^]_i_ rise (diastolic d[Ca^2+^]_i_/dt) were significantly different in HF SAN cells compared with CTRL SAN cells (Figure 1B, bottom right panels).

**Figure 2:**
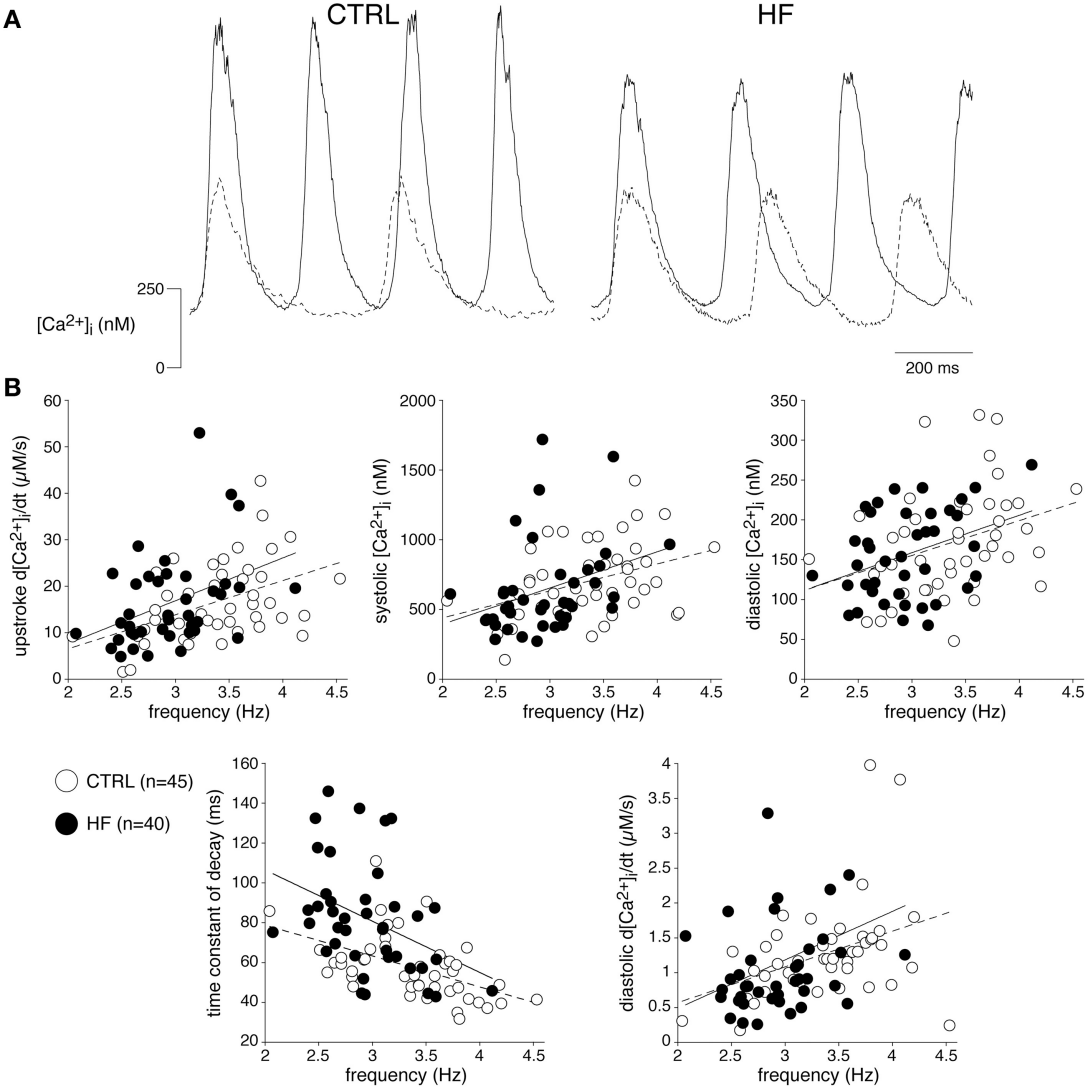
**The relationships between frequency and [Ca^2+^]_i_ transient parameters are not affected by HF**. **(A)** Typical [Ca^2+^]_i_ transients of a fast (solid line) and slow (dashed line) beating CTRL and HF SAN cell. **(B)** Scatter plot of [Ca^2+^]_i_ transient parameters of all cells measured vs. frequency. Solid and dashed lines: linear fits to HF and CTRL data, respectively.

In ventricular myocytes, [Ca^2+^]_i_ transient parameters are action potential duration (Weber et al., 2003) and frequency-dependent (Hussain et al., 1997; Dibb et al., 2007), while in the SAN an increase in late diastolic [Ca^2+^]_i_ rise was observed at higher beating rates (Joung et al., 2009). The role of the action potential duration in the observed slower [Ca^2+^]_i_ transient decay is likely limited, because the action potential duration in CTRL and HF SAN cells is similar (Verkerk et al., 2003). However, the decay of the [Ca^2+^]_i_ transient has a strong frequency dependency with a slower decay at slower frequencies (Hussain et al., 1997; Dibb et al., 2007). Thus, the slower [Ca^2+^]_i_ transient decay that we observed in HF SAN cells may be related to its slower intrinsic frequency. To test this hypothesis, we determined the relationship between frequency and [Ca^2+^]_i_ properties by plotting each of the five [Ca^2+^]_i_ transient parameters of every cell against its own frequency. The data have been fitted with linear regression lines (Figure 2). The linear fits to the CTRL as well as HF SAN cell data have all slopes significantly different from zero (see Table 2). However, neither the slopes nor the intercepts are significantly different between CTRL and HF for each of the five parameters (Table 2). Thus, while there are clear relationships between all five [Ca^2+^]_i_ transient parameters and frequency, these relationships do not differ significantly between CTRL and HF SAN cells. The ≈41% difference in [Ca^2+^]_i_ transient time constant of decay between HF and CTRL cells (Figure 1B) is larger than expected from the linear regression of the [Ca^2+^]_i_ transient time constant of decay vs. frequency relationship. Using the slope and intercept of the regression line for all cells, which amount to 151 (intercept) and −26 (slope), respectively, the difference in frequency between HF and CTRL cells explains only a ≈17% difference.

**Table 2:** **Frequency-dependency of [Ca^2+^]_i_ transient parameters in CTRL and HF SAN cells**.

	**CTRL (*n* = 45)**		**HF (*n* = 40)**	
	**Slope**	**Intercept**	**Slope**	**Intercept**
Upstroke d[Ca^2+^]_i_/dt	7.3 ± 2.2	−8.1 ± 7.5	9.2 ± 3.6	−10.7 ± 10.6
Systolic [Ca^2+^]_i_	194 ± 73	55 ± 250	260 ± 121	−124 ± 359
Time constant of decay	−16 ± 4	110 ± 14	−26 ± 10	158 ± 29
Diastolic [Ca^2+^]_i_	44 ± 18	25 ± 62	47 ± 20	18 ± 59
Diastolic d[Ca^2+^]_i_/dt	0.51 ± 0.19	−0.45 ± 0.66	0.70 ± 0.34	−0.90 ± 1.0

Regression lines as in Figure 2. Slope and intercept data are ± standard error. All slopes are significantly different from zero.

From these data we conclude that HF reduces the frequency and slows the Ca^2+^ transient decay of SAN cells, the latter partially explained by the slower frequency in HF SAN cells.

### HF does not affect the SR Ca^2+^ content of SAN cells

**Figure 3:**
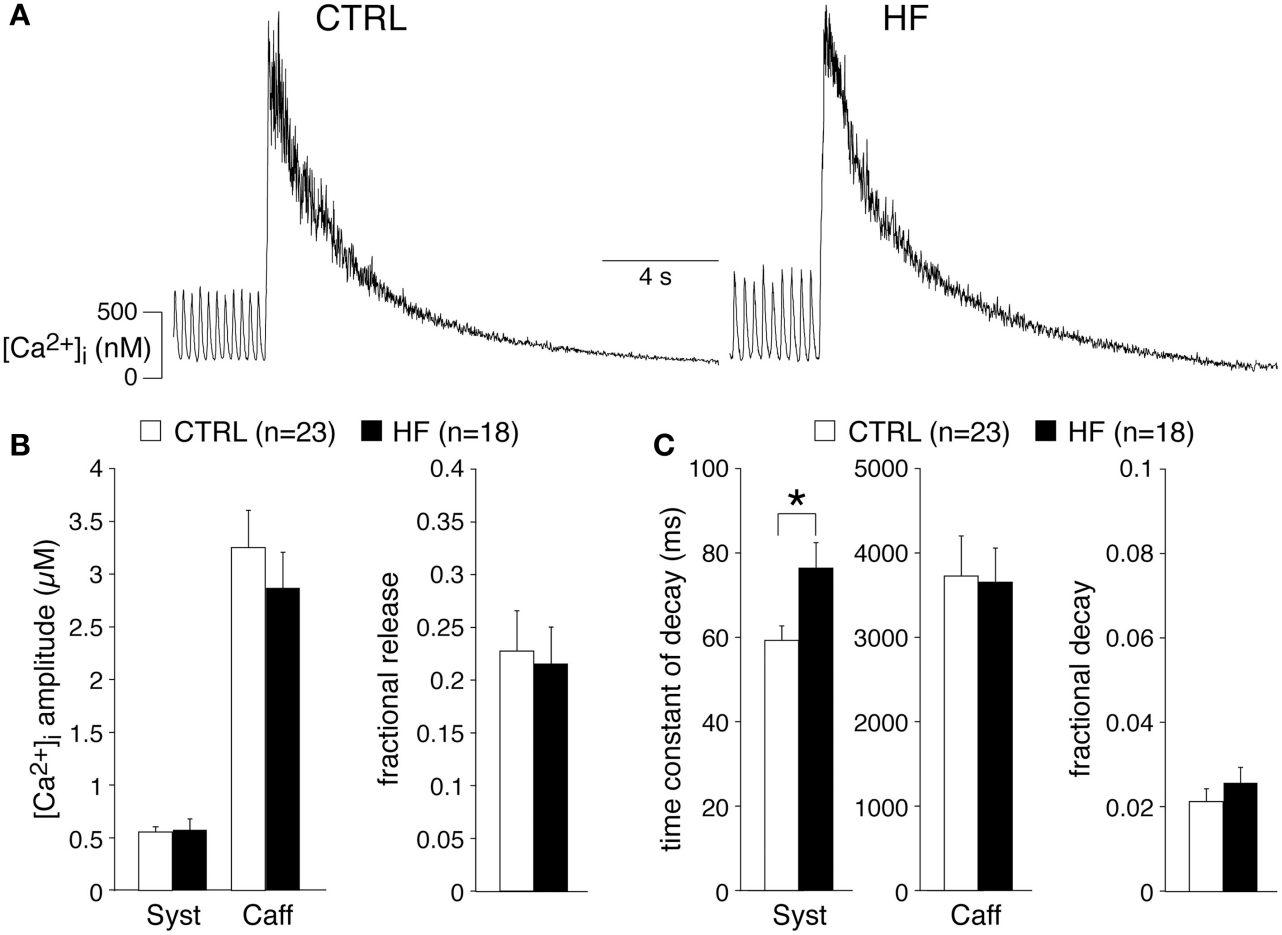
**HF does not affect the sarcoplasmic reticulum (SR) Ca^2+^ content of SAN cells. (A)** Typical examples of [Ca^2+^]_i_ transients induced by application of 20 mM caffeine in the presence of 5 mM NiCl_2_. **(B)** Average amplitudes of systolic (Syst) and caffeine-induced (Caff) [Ca^2+^]_i_ transients (left panel) and the average fractional release (right panel). **(C)** Average time constants of decay of the systolic and caffeine-induced [Ca^2+^]_i_ transients (left panels). Right: fractional contribution of mitochondrial Ca^2+^ uptake and sarcolemmal Ca-ATPase to the [Ca^2+^]_i_ transient decay. ^*^P < 0.05.

Our experiments demonstrate a reduced [Ca^2+^]_i_ transient decay rate, partially explained by a slower intrinsic frequency. The decline of the [Ca^2+^]_i_ transient is mainly due to uptake into the SR through SERCA and extrusion of Ca^2+^ via the sarcolemmal Na^+^–Ca^2+^ exchange (NCX), while mitochondrial Ca^2+^ uptake and sarcolemmal Ca-ATPase may contribute for a very small fraction (Bers, 2006b; Dibb et al., 2007). In ventricular myocytes, a slower decline of the [Ca^2+^]_i_ transient by downregulation of SERCA results in a reduced SR Ca^2+^ content (Bers, 2006b; Bers et al., 2006), while a slower decline of the [Ca^2+^]_i_ transient due to downregulation of the NCX may result in increased SR Ca^2+^ content (Bers, 2006b; Eisner et al., 2013). To gain insight into the relationship in SAN cells, we analyzed the amplitude of the normal spontaneous [Ca^2+^]_i_ transient as a fraction of the caffeine-evoked transient in the presence of 5 mM NiCl_2_, termed fractional release, which is an estimate of the proportion of the SR Ca^2+^ content released during each spontaneous transient. Figure 3A shows typical recordings in SAN cells from a CTRL and a HF rabbit. The averaged fractional release of CTRL and HF SAN cells did not differ significantly [0.23 ± 0.04 (*n* = 23) vs. 0.22 ± 0.03 (*n* = 18)] (Figure 3B). The caffeine-evoked transient in the presence of NiCl_2_ can also be used to determine the aforementioned contribution of mitochondrial Ca^2+^ uptake and sarcolemmal Ca-ATPase, because SERCA and NCX function is effectively by-passed by caffeine and Ni^2+^, respectively (Díaz et al., 2004). The time constant of decay of the caffeine-evoked [Ca^2+^]_i_ transient in the presence of 5 mM NiCl_2_ was 3728 ± 474 (CTRL, *n* = 23) vs. 3654 ± 406 ms (HF, *n* = 18) (Figure 3C), indicating that mitochondrial Ca^2+^ uptake and sarcolemmal Ca-ATPase were not affected in HF SAN cells compared to CTRL SAN cells. The relative contribution of the mitochondrial Ca^2+^ uptake and sarcolemmal Ca-ATPase in Ca^2+^ removal was calculated by the use of the time constants of decay of the systolic [Ca^2+^]_i_ transient (Figure 3C, left) and those of caffeine-evoked [Ca^2+^]_i_ transient in the presence of Ni^2+^ (Figure 3C, middle) (Díaz et al., 2004). The percentage that mitochondrial Ca^2+^ uptake and sarcolemmal Ca-ATPase contribute to the [Ca^2+^]_i_ transient decay was 2.1 ± 0.3 and 2.6 ± 0.4 in CTRL and HF SAN cells, respectively (Figure 3C, right).

From these data we conclude that HF neither affects the SR Ca^2+^ content nor the mitochondrial Ca^2+^ uptake and sarcolemmal Ca-ATPase activity of SAN cells.

### HF affects neither the I_NCX_ nor its [Ca^2+^]_i_ dependency

**Figure 4:**
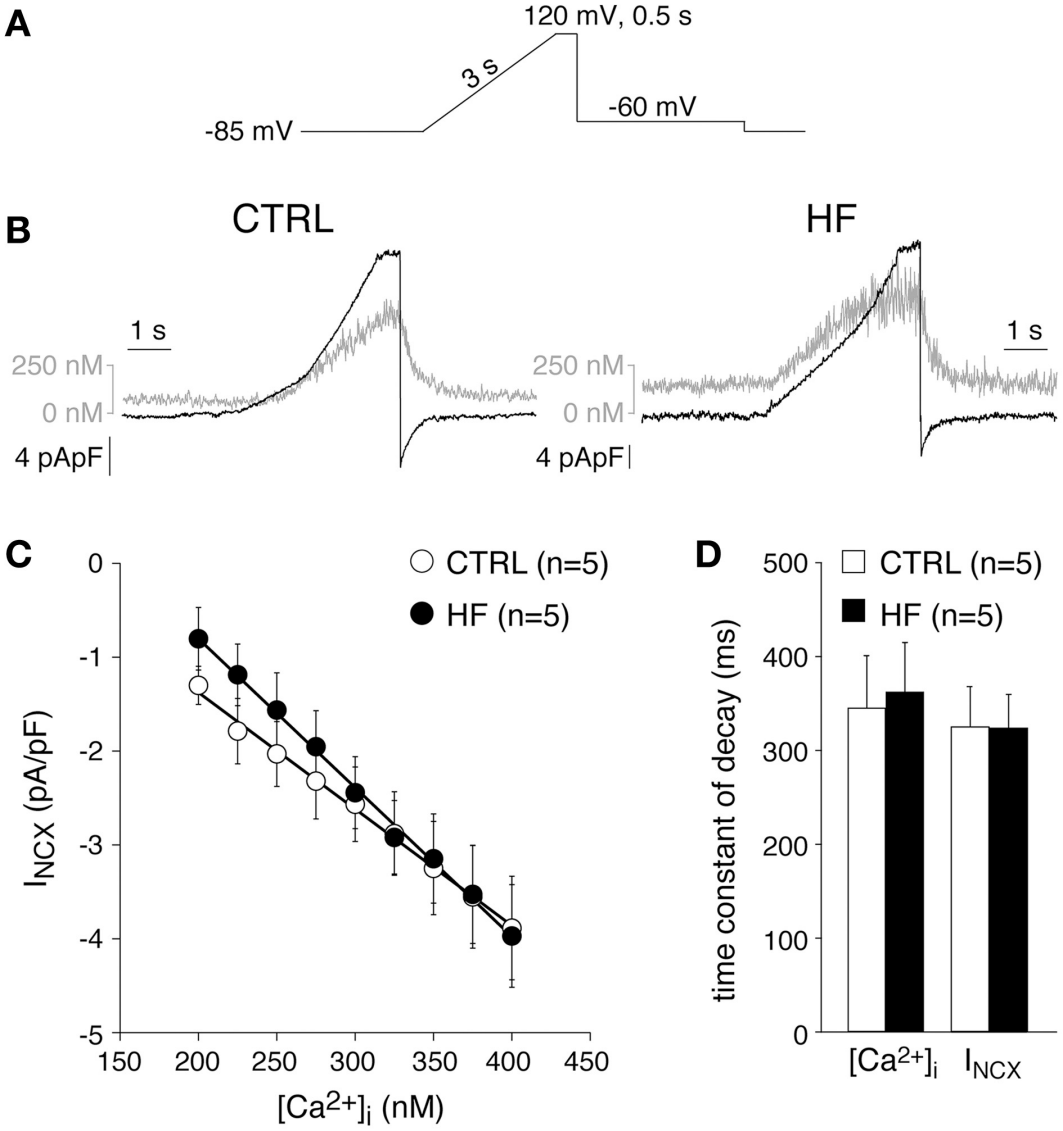
**HF affects neither the Na^+^–Ca^2+^ exchange current (I_NCX_) nor its [Ca^2+^]_i_ dependency. (A)** Voltage clamp protocol used. **(B)** Typical changes in [Ca^2+^]_i_ and I_NCX_ (gray and black traces, respectively) in response to the voltage clamp protocol of **(A)**. **(C)** Average I_NCX_-[Ca^2+^]_i_ relationships obtained during the repolarization-induced [Ca^2+^]_i_ decline. Solid lines: linear fits. **(D)** Average time constants of the [Ca^2+^]_i_ and I_NCX_ decline during the repolarizing step to −60 mV.

Previously, the I_NCX_ density was found to be similar in CTRL and HF SAN cells of rabbit (Verkerk et al., 2003). These results were obtained by measurements of Ni^+^-sensitive currents during a descending ramp, while the free [Ca^2+^]_i_ was buffered at 150 nM (Verkerk et al., 2003). HF may affect the relationship between [Ca^2+^]_i_ and I_NCX_ (Díaz et al., 2004). Therefore, we next determined the relationship between [Ca^2+^]_i_ and I_NCX_ as described previously by Weber et al. (2003). Figures 4A,B, show the protocol used and typical examples, respectively. A slowly depolarizing voltage ramp from −85 to 120 mV results in an increase in [Ca^2+^]_i_ due to Ca^2+^ entry through reverse mode NCX (Figure 4B). Upon repolarization to −60 mV, Ca^2+^ removal occurs (forward mode NCX) resulting in a [Ca^2+^]_i_ decline (Figure 4B, gray lines), which correlates well with the measured I_NCX_ (Figure 4B, black lines) (cf. Weber et al., 2003). Figure 4C shows the average I_NCX_-[Ca^2+^]_i_ relationships obtained during the repolarization-induced [Ca^2+^]_i_ decline in CTRL and HF SAN cells. The I_NCX_-[Ca^2+^]_i_ relationships are linear in the physiological range. Neither the I_NCX_ density nor the I_NCX_-[Ca^2+^]_i_ relationships differ significantly between HF and CTRL SAN cells (Figure 4C). In addition, the time constants of both the [Ca^2+^]_i_ and I_NCX_ decline during the repolarizing step to −60 mV, another measure of NCX function (Weber et al., 2003), do not differ significantly between CTRL and HF SAN cells (Figure 4D).

From these data we conclude that HF does not affect the I_NCX_ density nor the I_NCX_-[Ca^2+^]_i_ dependency. In addition, given the unaltered NCX and mitochondrial Ca^2+^ uptake and sarcolemmal Ca-ATPase activity of SAN cells, we conclude that the slower decay of the [Ca^2+^]_i_ transient is due to reduced SERCA activity.

### HF hardly affects [Ca^2+^]_i_ modulation by acethylcholine and noradrenaline

**Figure 5:**
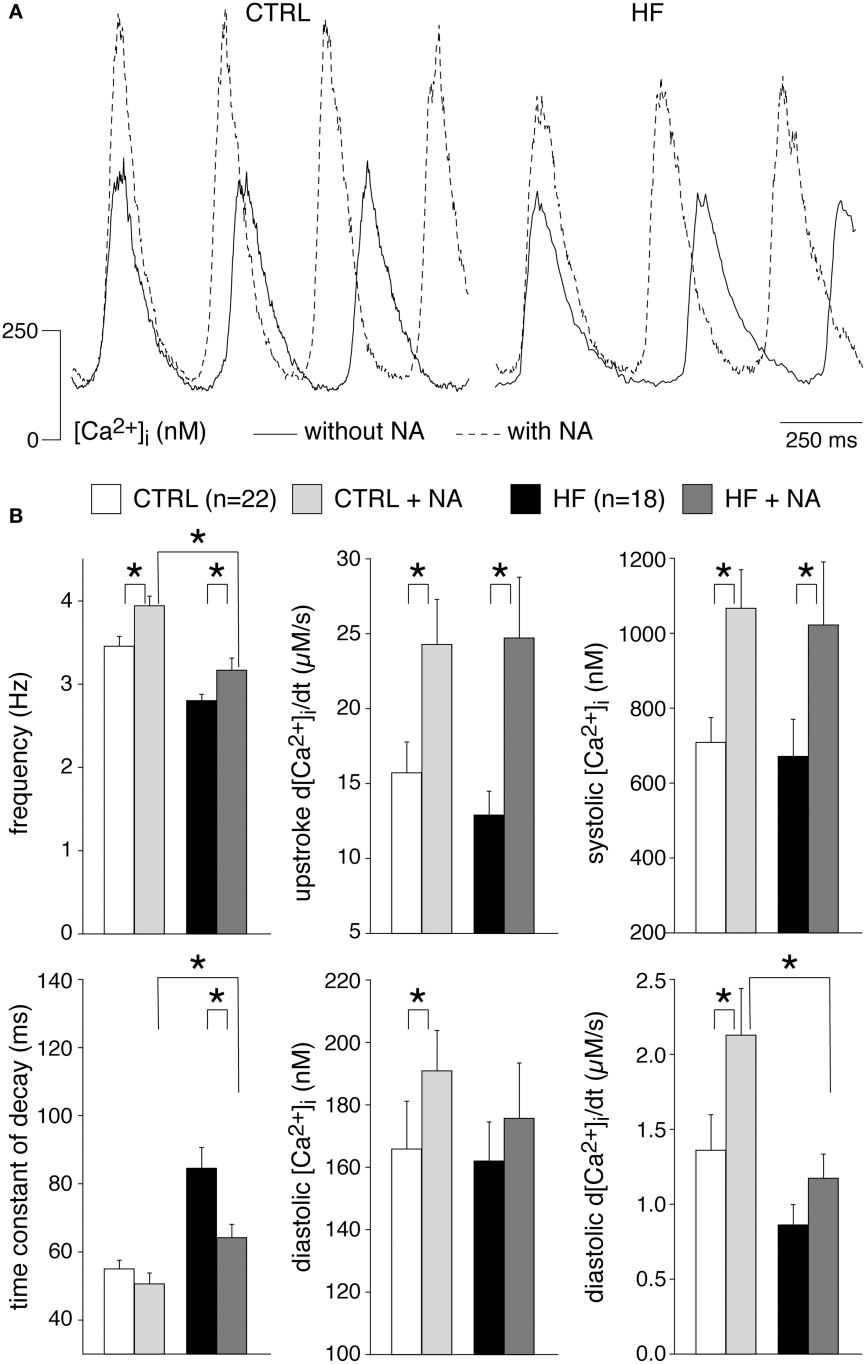
**Effects of 500 nM noradrenaline (NA) on the [Ca^2+^]_i_ transient parameters in CTRL and HF SAN cells. (A)** Representative [Ca^2+^]_i_ transients in absence (solid lines) and presence (dashed lines) of NA. **(B)** Average [Ca^2+^]_i_ transient characteristics in absence and presence of NA. ^*^P < 0.05.

[Ca^2+^]_i_ transients in SAN cells are importantly modulated by β-adrenergic (Rigg et al., 2000; Vinogradova et al., 2002a; Joung et al., 2009; van Borren et al., 2010) and muscarinic receptor (van Borren et al., 2010) stimulation. Most importantly, β-adrenergic stimulation increases the frequency and amplitude of [Ca^2+^]_i_ transients as well as local Ca^2+^ releases (LCRs) from the SR late during the diastolic depolarization (Vinogradova et al., 2002a; Joung et al., 2009; van Borren et al., 2010). Muscarinic receptor stimulation with acethylcholine decreases the frequency and amplitude of [Ca^2+^]_i_ transients and local Ca^2+^ releases from the SR (van Borren et al., 2010). Opthof et al. (2000) reported an increased sensitivity for acetylcholine in intact HF SAN preparations of rabbit, while Shinohara et al. (2010) found that intact SAN preparations of HF dogs were completely unresponsive to isoproterenol. Here, we tested the effects of β-adrenergic and muscarinic receptor stimulation on [Ca^2+^]_i_ transient parameters in CTRL and HF SAN cells. β-adrenergic stimulation with 500 nM noradrenaline significantly increased the frequency and the [Ca^2+^]_i_ transient parameters in both CTRL and HF SAN cells (Figure 5), except the time constant of decay, which was significantly decreased in the HF SAN cells. In presence of noradrenaline HF SAN cells still have a significantly lower frequency and slower decay phase compared to CTRL SAN cells (Figure 5B, left), while now also the late diastolic [Ca^2+^]_i_ rise is significantly lower in HF SAN cells (Figure 5B, bottom right).

**Figure 6:**
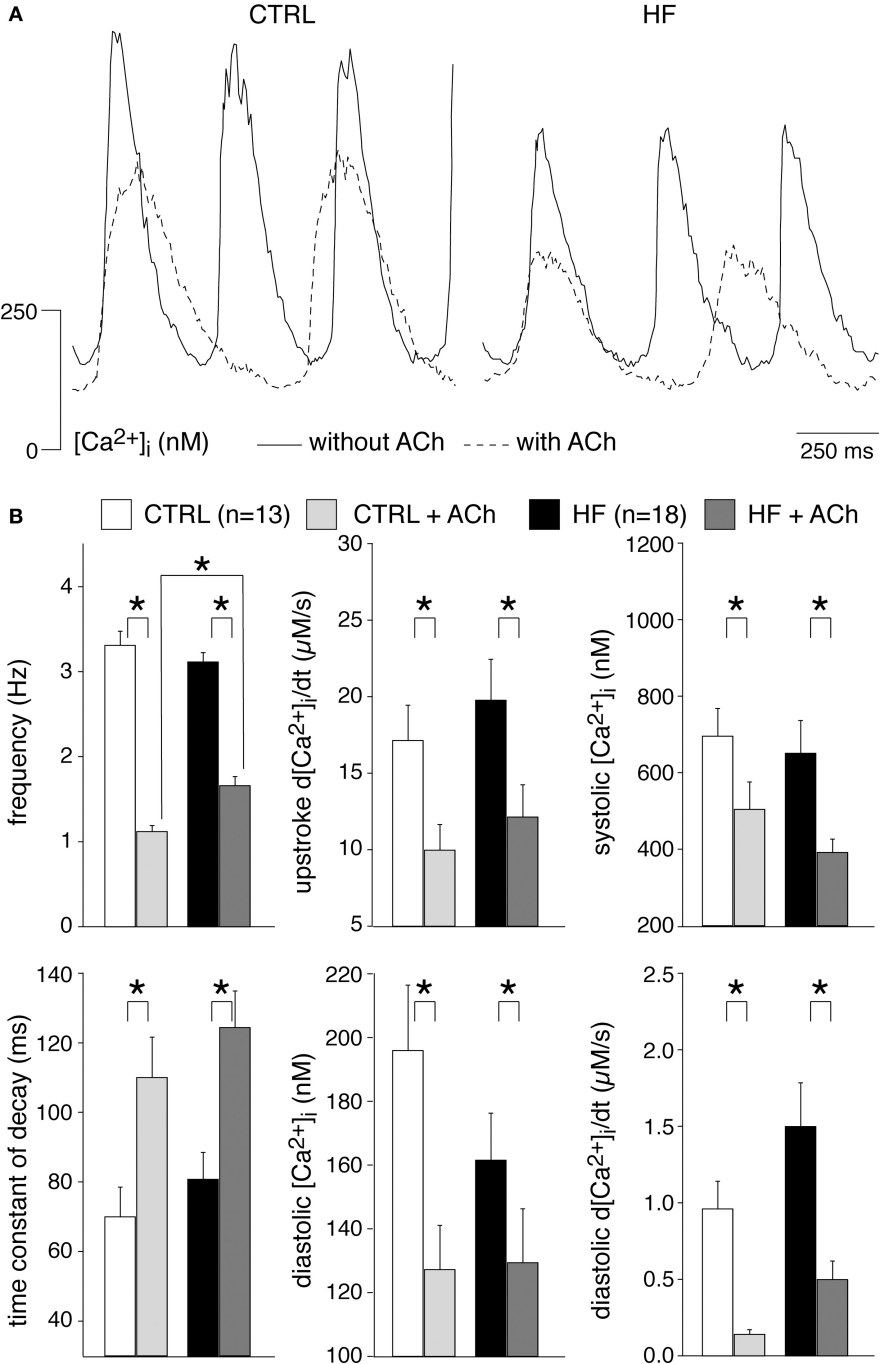
**Effects of 50 nM acetylcholine (ACh) on the [Ca^2+^]_i_ transient parameters in CTRL and HF SAN cells. (A)** Representative [Ca^2+^]_i_ transients in absence (solid lines) and presence (dashed lines) of ACh. **(B)** Average [Ca^2+^]_i_ transient characteristics in absence and presence of ACh. ^*^P < 0.05.

Muscarinic receptor stimulation with 50 nM acetylcholine significantly decreased the frequency and [Ca^2+^]_i_ transients parameters in both CTRL and HF SAN cells, with exception of the time constant of decay which was significantly increased in both groups of cells (Figure 6). In presence of acetylcholine, only the frequency differs significantly between HF and CTRL SAN cells.

From these data we conclude that HF does not affect importantly the [Ca^2+^]_i_ modulation by acethylcholine and noradrenaline. However, in presence of noradrenaline, HF SAN cells have a reduced late diastolic [Ca^2+^]_i_ rise compared to CTRL SAN cells and this might have implications for pacemaker activity formation during β-adrenergic stimulation.

### Effects of HF on the I_NCX_ during the diastolic depolarization phase

The [Ca^2+^]_i_ transients are longer in HF SAN cells, likely due to reduced SERCA activity. In general, it is assumed that this will promote NCX mediated Ca^2+^ extrusion, and thus will increase the I_NCX_-mediated net inward current during the diastolic depolarization (Ju and Allen, 1998; Sanders et al., 2006; Lau et al., 2011). Such an effect may, at least partially, counteract the previously observed decrease in I_f_-mediated inward current in our used model of HF (Verkerk et al., 2003). However, NCX function during the cardiac cycle is not only determined by [Ca^2+^]_i_, but also by the intracellular Na^+^ ([Na^+^]_i_) concentration, and the membrane potential (V_m_) (Blaustein and Lederer, 1999).

**Figure 7:**
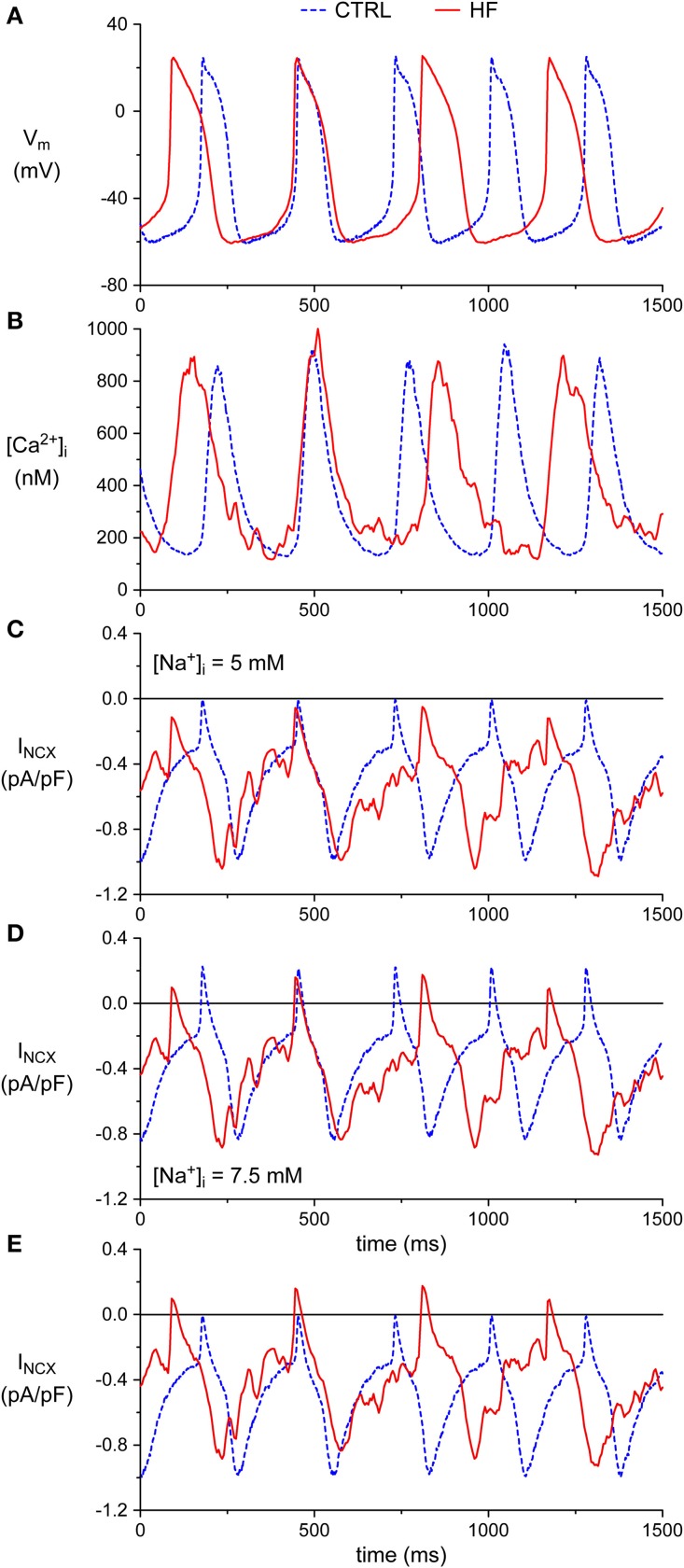
**Simultaneous action potential and [Ca^2+^]_i_ transient recordings from CTRL and HF SAN cells and associated numerical reconstruction of the I_NCX_**. **(A)** Recorded membrane potential (V_m_). **(B)** Recorded intracellular [Ca^2+^]_i_ concentration. **(C)** Reconstructed I_NCX_. **(D)** I_NCX_ upon elevation of the intracellular Na^+^ concentration ([Na^+^]_i_) to 7.5 mM. **(E)** Selection of the I_NCX_ of the CTRL SAN cell of **(C)** ([Na^+^]_i_ of 5 mM) and HF SAN cell of **(D)** ([Na^+^]_i_ of 7.5 mM).

Next, we recorded action potentials (APs) simultaneously with [Ca^2+^]_i_ in combined [Ca^2+^]_i_ and patch clamp experiments, and calculated I_NCX_ during SAN APs using the measured [Ca^2+^]_i_ and V_m_. Combined [Ca^2+^]_i_ and patch clamp experiments were performed in 7 CTRL and 5 HF SAN cells. The average [Ca^2+^]_i_ transient and AP parameters recapitulate the CTRL and HF SAN cell phenotype of the present paper (Figure 1) and of our previous study (Verkerk et al., 2003; Figure 1), although the differences in frequency and diastolic depolarization rate between CTRL and HF SAN cells did not reach the level of statistical significance due to the small number of cells (data not shown). Figures 7A,B, show a selected AP and [Ca^2+^]_i_ transient of a CTRL and HF SAN cell, which closely represent the mean differences in [Ca^2+^]_i_ transient and AP properties. Figure 7C shows the reconstructed I_NCX_, based on the simultaneously recorded Ca^2+^ transients and APs of Figures 7A,B. In the CTRL SAN cell, I_NCX_ is inward for the entire AP, with a small amplitude early in the AP and a maximal amplitude late during repolarization. The I_NCX_ declined during the diastolic depolarization phase, consistent with the decrease in [Ca^2+^]_i_. In HF, I_NCX_ during the AP is close to that of the CTRL SAN cells, except that the amplitude is slightly larger during the diastolic depolarization. This is likely due to the slightly higher [Ca^2+^]_i_ in the diastolic depolarization phase, since the APs were almost identical and an identical [Na^+^]_i_ concentration of 5 mM was used for CTRL and HF SAN cells in both the pipette solution and calculations. Figure 7D extends our analysis to an increased [Na^+^]_i_, as frequently observed in ventricular myocytes of our used HF model (Baartscheer et al., 2003b). Elevation of the [Na^+^]_i_ by 50% to 7.5 mM, in line with the observations of Baartscheer et al. (2003b), decreased the diastolic I_NCX_ in both CTRL and HF SAN cells. Interestingly, I_NCX_ may become even shortly outward early during the AP. Figure 7E shows the I_NCX_ of the CTRL SAN cell with 5 mM [Na^+^]_i_ and the I_NCX_ of the HF SAN cell with 7.5 mM [Na^+^]_i_. It is evident that under these conditions I_NCX_ in HF SAN cell is close to that of the CTRL SAN, except for the outward I_NCX_ in the HF SAN cell. Similar results are obtained with the NCX model equations of Faber and Rudy (2000) (data not shown).

From these data we conclude that the decreased [Ca^2+^]_i_ transient decay in HF may result in slightly increased I_NCX_ during the diastolic depolarization phase, but that this effect is counteracted by a HF-induced increase in [Na^+^]_i_.

## Discussion

In the present study, we characterized the [Ca^2+^]_i_ cycling of single SAN cells isolated from CTRL rabbits and rabbits with volume and pressure overload-induced HF. The mechanisms behind [Ca^2+^]_i_ cycling in ventricular myocytes are diverse and complex (for reviews, see Guatimosim et al., 2002; Eisner et al., 2005; Bers, 2006b; Neef and Maier, 2013). In short, the [Ca^2+^]_i_ transient is triggered by Ca^2+^ influx through sarcolemmal Ca^2+^ channels, which results in release of Ca^2+^ from the SR via ryanodine-2 (RyR2) channels. This so-called Ca^2+^-induced Ca^2+^ release (CICR) is importantly modulated by T-tubuli organization, with co-localization of I_Ca,L_ and RyR2 channels (Øyehaug et al., 2013), and the open probability of RyR2 channels (Guatimosim et al., 2002). The amplitude of the [Ca^2+^]_i_ transient depends importantly on the SR Ca^2+^ content (Bassani et al., 1992; Trafford et al., 2000, 2001; Díaz et al., 2004; Dibb et al., 2007; Briston et al., 2011). The decline of the [Ca^2+^]_i_ transient is mainly due to SR Ca^2+^ reuptake through SERCA and extrusion of Ca^2+^ via the NCX. Because NCX and SERCA activity compete during the decline of the [Ca^2+^]_i_ transient, any change in SERCA function indirectly affects NCX contribution and vice versa, resulting in changes in the Ca^2+^ content of the SR and consequently [Ca^2+^]_i_ transient amplitudes (Eisner et al., 2005; Bers, 2006a; Neef and Maier, 2013). The diastolic Ca^2+^ concentration is regulated by the [Ca^2+^]_i_ transient decline, especially during rapid pacing (Laurita et al., 2003), and leak of RyR2 channels (Neef and Maier, 2013). Both a slower [Ca^2+^]_i_ transient decay and increased RyR2 channel leak results in elevation of the diastolic Ca^2+^ concentration. SAN cells exhibit also another [Ca^2+^]_i_ transient characteristic, i.e., rise of the diastolic [Ca^2+^]_i_, which is a key signature of pacemaking by the Ca^2+^ clock (for review, see Maltsev and Lakatta, 2008; Lakatta et al., 2010; Joung et al., 2011).

HF in ventricular myocytes may affect many [Ca^2+^]_i_ transient characteristics. It is reported that HF decreases the upstroke velocity of the [Ca^2+^]_i_ transient by T-tubuli disorganization (Øyehaug et al., 2013) and increased open probability of RyR2 channels (Guatimosim et al., 2002). An increase of RyR2 open probability, as may occur during HF in ventricular myocytes, also leads to an increased amount of Ca^2+^ sparks, resulting in an increased diastolic [Ca^2+^]_i_ concentration and reduced SR Ca^2+^ content since more Ca^2+^ is pumped out of the cell by the NCX (Neef and Maier, 2013). Furthermore, HF in ventricular mycoytes typically slows the [Ca^2+^]_i_ transient decay due to reduced SERCA function. Such reduced Ca^2+^ uptake by SERCA, in combination with a frequently observed upregulation of the NCX and consequently increased Ca^2+^ efflux, will reduce the SR Ca^2+^ content and systolic Ca^2+^ transient amplitude (for reviews, see Bers, 2006b; Eisner et al., 2013; Neef and Maier, 2013). Of note, the diastolic [Ca^2+^]_i_ concentration is frequently elevated by HF, due to the slower [Ca^2+^]_i_ transient decay and increased RyR2 channel leak.

We found that HF SAN cells have (1) a reduced frequency of spontaneous [Ca^2+^]_i_ transients, (2) a slower [Ca^2+^]_i_ transient decay, and (3) a reduced diastolic [Ca^2+^]_i_ rise during β-adrenergic stimulation, compared to CTRL SAN cells. Combined action potential and [Ca^2+^]_i_ measurements demonstrated that the decreased [Ca^2+^]_i_ transient decay in HF SAN cells may result in slightly increased I_NCX_ during the diastolic depolarization phase, but that this effect is counteracted by HF-induced increase in [Na^+^]_i_.

### Intrinsic cycle length is prolonged in HF SAN cells

We found a lower frequency of spontaneous [Ca^2+^]_i_ transients in HF SAN cells, indicating that intrinsic cycle length was prolonged in HF SAN cells (Figure 1). This finding agrees with previously findings in intact SAN (Opthof et al., 2000) and single SAN cells (Verkerk et al., 2003) of rabbit. The frequency of the intrinsic cycle length based on the [Ca^2+^]_i_ transients was 14% slower in HF SAN cells (Figure 1) and this percentage closely matches the increased intrinsic cycle length (15%) of our previous study with the same rabbit HF model and measured with perforated patch clamp methodology (Verkerk et al., 2003).

We found that the frequency of the spontaneous [Ca^2+^]_i_ transients in both CTRL and HF SAN cells influenced various [Ca^2+^]_i_ transient characteristics, including diastolic and systolic [Ca^2+^]_i_ concentrations, systolic [Ca^2+^]_i_ rise and decay, and late diastolic [Ca^2+^]_i_ rise (Figure 2, Table 2). All parameters, except the [Ca^2+^]_i_ transient time constant of decay, increased upon increased frequencies. The frequency dependency was not significantly different in HF compared to CTRL SAN cells (Figure 2, Table 2). Nevertheless, the frequency dependency relationships might have influenced some principal findings because spontaneous activity is lower in HF SAN cells. The relative small difference in intrinsic cycle length of CTRL and HF SAN cells, the rather large variation between cells, and the modest steepness of the frequency-dependencies (Table 2, Figure 2) may all have contributed to the absence of significant differences in frequency dependency between HF and CTRL SAN cells.

Recently, Herrmann et al. elegantly solved the problem of intrinsic rate differences in SAN cells by using electrical field stimulation (Herrmann et al., 2013). Using this approach, they investigated the contribution of the murine sodium-calcium exchanger protein NCX1 to cardiac pacemaking in transgenic mice selectively lacking NCX1 in the cardiac pacemaking and conduction system. Among other things, they found that [Ca^2+^]_i_ transients measured during electrical field stimulation were of smaller magnitude and decelerated kinetics in NCX1 knockout cells.

### [Ca^2+^]_i_ transient characteristics in HF SAN cells

#### [Ca^2+^]_i_ transient under basal conditions

We observed that HF results in a slower [Ca^2+^]_i_ transient decay (Figure 1). The [Ca^2+^]_i_ transient time constant of decay was increased by 41% in HF cells, which is larger than expected from the [Ca^2+^]_i_ transient time constant of decay vs. frequency relationship that explains only a ≈17% difference. HF affected neither the I_NCX_ density and I_NCX_-[Ca^2+^]_i_ relationship (Figure 4) nor the mitochondrial Ca^2+^ uptake and sarcolemmal Ca-ATPase (Figure 3), indicating that downregulation of SERCA activity also contributes to the slower [Ca^2+^]_i_ transient decay. Any contribution of (changes in) T-tubular organization, sarcolemmal calcium currents, and action potential duration can be ruled out because T-tubuli are absent in rabbit SAN cells (Masson-Pévet et al., 1979), and I_Ca,T_ and I_Ca,L_ densities as well as action potential duration are unaffected in HF rabbit SAN cells (Verkerk et al., 2003).

HF in SAN cells did not affect the diastolic and systolic [Ca^2+^]_i_ concentrations, systolic [Ca^2+^]_i_ rise, late diastolic [Ca^2+^]_i_ rise (Figure 1), and SR Ca^2+^ content (Figure 3). The unaffected diastolic Ca^2+^ concentration, at first sight, might appear to be inconsistent with the decrease of the [Ca^2+^]_i_ transient decline. It should be noted, however, that HF SAN cells have a lower frequency due to their longer diastolic depolarization phase (Verkerk et al., 2003), which leaves more time for Ca^2+^ reuptake and/or removal. The unchanged slope of the LDCAE, associated with LCRs and Ca^2+^ sparks, suggests that RyR2 open probability is not affected in HF SAN cells. Our finding contrasts findings in a canine model of rapid pacing-induced heart failure, where it was found, using isolated right atrial preparations, that LDCAE was reduced (Shinohara et al., 2010). Differences in HF model, species and preparations might contribute to the contrasting findings.

The unaltered SR Ca^2+^ content agrees with the unaffected [Ca^2+^]_i_ transient amplitude in HF SAN cells, but is somewhat surprising given that reduced SERCA activity is supposed to result in a lower SR Ca^2+^ content. The latter is due because NCX and SERCA activity compete for Ca^2+^ during the [Ca^2+^]_i_ transient decline, and reduced SERCA function thus indirectly favors greater Ca^2+^ efflux via the NCX (for reviews, see Guatimosim et al., 2002; Eisner et al., 2005; Bers, 2006b; Neef and Maier, 2013). We observed a similar SR Ca^2+^ content in HF and CTRL SAN cells despite the slower [Ca^2+^]_i_ transient decline in HF SAN cells. This suggests a compensatory increase in Ca^2+^ influx. I_Ca,T_ and I_Ca,L_ densities were not affected in HF SA node cells (Verkerk et al., 2003), but we cannot exclude that the longer diastolic depolarization phase in HF SAN cells (Verkerk et al., 2003) may result in a larger background Ca^2+^ influx via L-type Ca^2+^ channels (Verheijck et al., 1999). Alternatively, increased [Na^+^]_i_ as occurs during HF may promote Ca^2+^ influx via reversed NCX activity (Despa et al., 2002). Indeed, by reconstruction of the I_NCX_, based on action potentials and simultaneously measured [Ca^2+^]_i_ transients, we found that the I_NCX_ became outward, thus resulting in Ca^2+^ influx, early during the AP (Figures 7D,E) under conditions of elevated [Na^+^]_i_ as occur during HF.

#### [Ca^2+^]_i_ transient during β-adrenergic and muscarinic receptor stimulation

We found that the β-adrenergic and muscarinic receptor stimulation was hardly affected by HF. In both CTRL and HF SAN cells, acetylcholine significantly decreased the frequency and [Ca^2+^]_i_ transients parameters, with exception of the time constant of decay which was significantly increased in both groups of cells (Figure 6). The ACh induced effects were largely similar in CTRL and HF SAN cells. Noradrenaline, on the other hand, significantly increased the frequency and most [Ca^2+^]_i_ transient parameters in both CTRL and HF SAN cells (Figure 5), leading to preserved [Ca^2+^]_i_ transient differences between the CTRL and the HF SAN cells. However, in presence of noradrenaline the late diastolic [Ca^2+^]_i_ rise was significantly lower in HF SAN cells compared to HF SAN cells. The lower late diastolic [Ca^2+^]_i_ rise in HF SAN cells in presence of noradrenaline is in agreement with findings in canine right atrial preparations by Shinohara et al. (2010).

### Implication of HF-induced [Ca^2+^]_i_ transient changes in pacemaker activity

Overall, the effects of HF on [Ca^2+^]_i_ transients in rabbit SAN cells were modest. The decrease in frequency of the spontaneous [Ca^2+^]_i_ transient in HF SAN cells is completely explained by the previously observed slower intrinsic cycle length (Verkerk et al., 2003). The slower [Ca^2+^]_i_ transient decay will promote Ca^2+^ transport across the sarcolemma by the NCX (Bers et al., 2006), an electrogenic process that delivers inward current at diastolic potentials. The observed slower [Ca^2+^]_i_ transient decay may thus result in increased I_NCX_ during the diastolic depolarization phase, thereby partially counteracting the previously observed decrease in I_f_-mediated inward current in HF (Verkerk et al., 2003). Indeed, reconstructed I_NCX_, based on the simultaneously recorded Ca^2+^ transients and APs, demonstrated a slightly larger amplitude during the diastolic depolarization phase (Figure 7C). However, when we incorporated an increased [Na^+^]_i_, as frequently observed in ventricular myocytes, including those from our rabbit HF model (Baartscheer et al., 2003b), the I_NCX_ in the diastolic depolarization phase is close to that of the CTRL SAN (Figure 7E). Thus, the slower [Ca^2+^]_i_ transient decay in HF has likely a limited role in the slower intrinsic cycle length in HF SAN cells. The slope of late diastolic [Ca^2+^]_i_ elevation, associated with localized Ca^2+^ releases (LCRs) or Ca^2+^ sparks (Bogdanov et al., 2006; Maltsev et al., 2006; Joung et al., 2009, 2010; van Borren et al., 2010), was unaffected under basal conditions, suggesting that the Ca^2+^ clock is not the primary cause of the slower intrinsic cycle length in HF SAN cells. Considering the importance of late diastolic [Ca^2+^]_i_ rise in setting pacemaker activity, however, the lower late diastolic [Ca^2+^]_i_ rise in HF SAN cells in presence of noradrenaline suggest that an impaired increase in intrinsic frequency under such conditions may be related to reduced Ca^2+^ clock function.

### Limitations

We used indo-1 to measure [Ca^2+^]_i_, because this ratiometric indicator is less prone to cell contractions and loss of dye. However, indo-1 had an adverse effect on cell viability and rendered many smaller cells quiescent (Lancaster et al., 2004). We cannot rule out the possibility that indo-1 has affected the principal findings, but we assume that its effect is limited because the differences in spontaneous activity of CTRL and HF SAN cells in our present study are highly similar to the differences that we observed with perforated patch clamp measurements (Verkerk et al., 2003).

In our study, we used the complete SAN for cell isolation. However, the SAN is not homogeneous in its composition. This seems valid for membrane currents (Boyett et al., 2000), but also the expression of several calcium handling proteins varies across the node (Musa et al., 2002; Lancaster et al., 2004), all of which are expressed at a lower level in the center of the SAN compared with the periphery, although such findings are debated (Lyashkov et al., 2007). Cells from the center and periphery may differ in cell capacitance. We exclude cell location-dependency as an explanation for our principal findings because cell capacitance did not differ between control and HF.

We reconstructed I_NCX_ based on simultaneous measurements of [Ca^2+^]_i_ and spontaneous action potentials as well as the [Na^+^]_i_ of 5 and 7.5 mM used in our pipette solution and found in our model of HF previously (Baartscheer et al., 2003b). Data on [Na^+^]_i_ in SAN cells are extremely sparse and we were not succesful in [Na^+^]_i_ measurements with benzofuran isophthalate (SBFI) ourselves. However, the used 5 mM [Na^+^]_i_ is close to the concentrations of 4.5 and 4.0 mM in rabbit multicellular SAN preparations and single SAN cells, respectively, found by Choi et al. (1999). In our reconstructions, we used the Lindblad et al. (1996) equations for I_NCX_. We could not make use of I_NCX_ equations from more recent models, in particular the rabbit SAN cell models by Maltsev and Lakatta (2010b) and Severi et al. (2012), because these require data on the concentration of Ca^2+^ in a sarcolemmal subspace rather than global [Ca^2+^]_i_.

## Conclusions

In our rabbit model of HF, SAN cells have reduced SERCA activity and reduced intrinsic frequency, both resulting in a slower Ca^2+^ decay. The decreased [Ca^2+^]_i_ transient decay in HF SAN cells may result in slightly increased I_NCX_ during the diastolic depolarization phase, but this effect is likely counteracted by HF-induced increase in [Na^+^]_i_. Reduced late diastolic [Ca^2+^]_i_ rise during β-adrenergic stimulation may contribute to the impaired increase in frequency under this condition in HF SAN cells.

### Conflict of interest statement

The authors declare that the research was conducted in the absence of any commercial or financial relationships that could be construed as a potential conflict of interest.
